# Node-Level Resilience Loss in Dynamic Complex Networks

**DOI:** 10.1038/s41598-020-60501-9

**Published:** 2020-02-27

**Authors:** Giannis Moutsinas, Weisi Guo

**Affiliations:** 1 School of Aerospace Transport and Manufacturing, Cranfield University, Bedford, United Kingdom; 2 The Alan Turing Institute, London, United Kingdom

**Keywords:** Applied mathematics, Applied mathematics, Complex networks, Complex networks

## Abstract

In an increasingly connected world, the resilience of networked dynamical systems is important in the fields of ecology, economics, critical infrastructures, and organizational behaviour. Whilst we understand small-scale resilience well, our understanding of large-scale networked resilience is limited. Recent research in predicting the effective network-level resilience pattern has advanced our understanding of the coupling relationship between topology and dynamics. However, a method to estimate the resilience of an individual node within an arbitrarily large complex network governed by non-linear dynamics is still lacking. Here, we develop a sequential mean-field approach and show that after 1-3 steps of estimation, the node-level resilience function can be represented with up to 98% accuracy. This new understanding compresses the higher dimensional relationship into a one-dimensional dynamic for tractable understanding, mapping the relationship between local dynamics and the statistical properties of network topology. By applying this framework to case studies in ecology and biology, we are able to not only understand the general resilience pattern of the network, but also identify the nodes at the greatest risk of failure and predict the impact of perturbations. These findings not only shed new light on the causes of resilience loss from cascade effects in networked systems, but the identification capability could also be used to prioritize protection, quantify risk, and inform the design of new system architectures.

## Introduction

Organized behaviour in economics^1^, infrastructure^2^, ecology^3^, biology^4^, and human society^5^ often involve large-scale networked systems, coupling together relatively simple dynamics to achieve complex behaviour. A critical part of the organized behaviour is the ability for a system to be resilient - the ability to retain original functionality after a perturbation^6^ or failure. When failures lead to disconnections, traditional robustness measures only consider topological changes, e.g. random removals to giant component collapse^7^. Yet, we know that the dynamics can play an important role, and often systems fail long before they are disconnected, e.g. connected components can lose desirable functionality due to cascade effects.

As we will see in the two case studies, the definition of resilience depends a lot on the exact system that is being studied.

## Background

**Figure 1:**
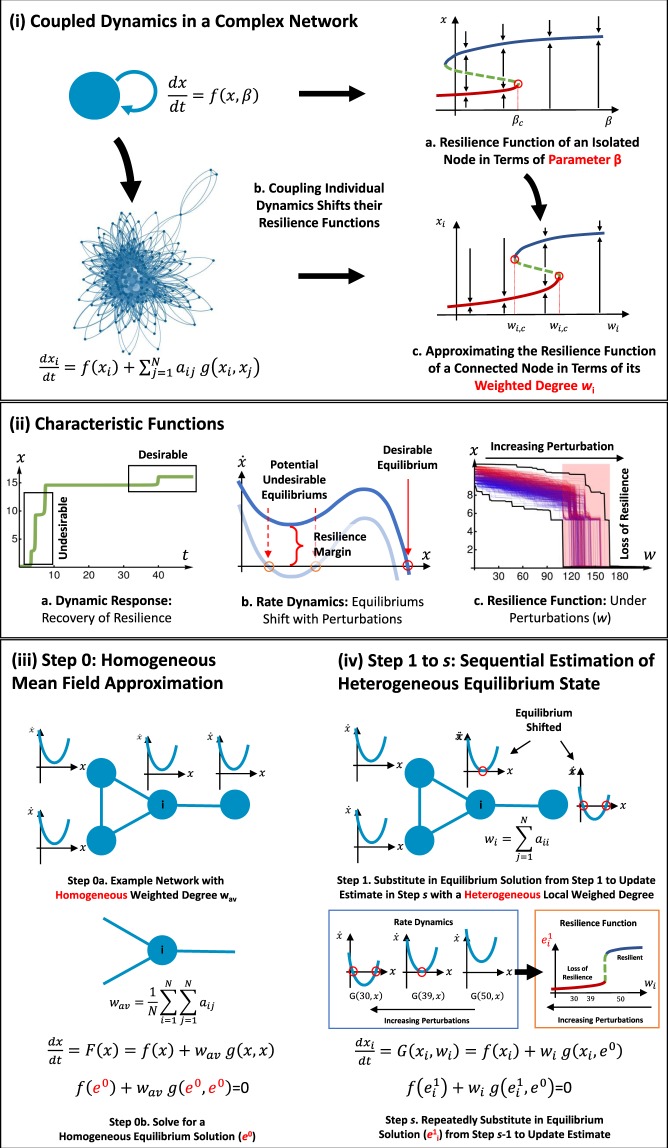
Estimating Node Level Resilience in Complex Networks. Each networked system is interested in a performance metric *x*, which is affected by disturbances. Desirable behaviour is contextual to the application (e.g. high *x* is the designed desirable operating regime). **(i)** Problem Definition - coupled dynamics in a complex network, where each node is governed a self-dynamic *f*(. ) and a coupling dynamic *g*(. ). This is connected together through a complex network. Individual resilience is sensitive to *β*, and connected resilience is sensitive to the topological measure *w*_*i*_. **(ii)** Characteristic Functions - (a) **dynamic response***x*(*t*) shows how a system or node can recover to desirable *x* value (resilience behaviour); (b) **rate dynamics**$\dot{x}(x)$ gives desirable and potentially undesirable equilibrium solutions, which change with perturbations; and (c) **resilience function***x*(*w*) describes how perturbations in the network property *w* cause unrecoverable collapse (loss in resilience). **(iii-iv)** **Method Overview**: Step 1: mean field approximation using weighted degree to estimate the **homogeneous** equilibrium solution *e*^0^ at all nodes. Step 2 to *s*: sequential substitution of equilibrium solution ${e}_{i}^{s-1}$ into *x*_*j*_ to estimate the **heterogeneous** equilibrium solution ${e}_{i}^{n}$.

For the example illustrated in Fig. 1i, a change in circumstance (represented by control parameter *β* in Eq. 1) can shift behaviour from a desirable (blue) to an undesirable (red) state. The definition of desirable state is application specific. The system cannot always bounce back to this desirable state and this is defined as a loss in resilience. Over the last few decades, practitioners have built up a strong understanding of each individual subsystem’s functional resilience. For example, a simple one-dimensional subsystem can be described by how the variable *x* changes:

1 $$\frac{dx}{dt}=f(x,\beta ),$$

where at equilibrium *f*(*x* = *e*, *β*) = 0 and $\frac{{\rm{d}}f}{{\rm{d}}x}{| }_{x=e} < 0$ maps to the resilience function *x*(*β*) given in Fig. 1i-a.

Whilst many physical, biological, ecological, social, and engineering systems have subsystems that can be described by Eq. 1, we do not have tractable understanding of the resilience function in large-scale networked dynamics (see Fig. 1i-b):

2 $$\frac{d{x}_{i}}{dt}=f({x}_{i})+\mathop{\sum }\limits_{j}^{N}{a}_{ji}g({x}_{i},{x}_{j}),$$

where each subsystem (node) *i*’s behaviour is described by a self-dynamic *f*(⋅) and a coupling dynamic *g*(⋅) with node *j* via the connectivity matrix *A*_*ij*_. We rewrite system 2 in the compact form:

3 $$\dot{X}=F(X),$$

where $F:{{\mathbb{R}}}^{N}\to {{\mathbb{R}}}^{N}$ defined by Eq. 2.

In general, we do not know very well how functional resilience maps to the topological resilience (e.g. properties of *A*_*ij*_) in connected ecosystems. Whilst tractable solutions exist for phase synchronization dynamics^8^ (e.g. Master Stability Function), general solutions do not exist for the non-linear dynamics that exist in many application areas set out in^9^. Indeed, recent research have begun to address this by mapping the overall effective dynamics of a networked system to its topological structure and individual dynamics^10^:

$${\dot{x}}_{{\rm{eff}}}({\beta }_{{\rm{eff}}},{x}_{{\rm{eff}}}),$$

where *x*_eff_ yields the effective mean network dynamics and *β*_eff_ captures effective aspects of the network topology. This work has been extended to consider negative interactions^11^, noise effects^12^, attack strategies^13^, and applied to critical infrastructure areas^14^. Other network level predictions using dimension reduction techniques have also been developed to yield similar insights^15^. However, we still do not understand the resilience and dynamics of individual nodes. As we will show, many systems can exhibit a common network-level effective dynamic, but have different node level dynamics. The precision to identify the resilience function at the node-level is sorely needed in all application domains in order to inform ground operations (e.g. prioritize conservation in ecology, enhance monitoring in infrastructures).

## Approach and Methodology

To answer this question, this paper presents a sequential estimation approach. This enables us to understand how network topology affects the resilience function of a node (see Fig. 1i-c). As an overview to the coupled dynamical system, we show how 3 key characteristic functions map to each other (see Fig. 1ii). First, we show that the dynamic response *x*(*t*) of the whole networked system or an individual node, can have a context dependent desirable and undesirable operating state. The dynamic response describes if the system can bounce back from undesirable to a desirable state over time. Second, we show that the rate dynamics $\dot{x}(x)$ defines the equilibrium states of the system, where there are desirable equilibrium and potentially undesirable ones (formation of which depends on dynamics, network, and perturbations). Finally, we show that by understanding the aforementioned dynamics, we can predict the resilience function of the whole system and each node as a function of perturbations *x*(*w*).

### Assumptions

For our method to work we assume the following:

1. The functions *f* and *g* are twice differentiable and there exists $R\in {{\mathbb{R}}}^{+}$ such that for all for all ∣*x*∣, ∣*y*∣ > *R* and $w\in {{\mathbb{R}}}^{+}$, (*f*(*x*) + *wg*(*x*, *y*)) *x* < 0.
2. The weights are independent and identically distributed random variables, with positive mean.
3. The graph has low degree correlation.

The necessity of the assumptions and the effect their violation has are discussed in the SI.

### Sequential estimation

The proposed framework utilizes an initial homogeneous mean field estimation (Fig. 1iii) to drive sequential substitution and evaluation of heterogeneous resilience at each node (Fig. 1iv). We also quantify the complexity of the algorithm at each step.

**Step 0:** First, we calculate a mean field approximation of the system. By using either a homogeneous average degree ${w}_{{\rm{av}}}=\frac{1}{N}{\sum }_{i}^{N}{\sum }_{j}^{N}{a}_{ij}$ or a weighted average degree

$${w}_{{\rm{av}}}=\frac{\left\langle {w}_{{\rm{out}}}\ {w}_{{\rm{in}}}\right\rangle }{\left\langle {w}_{{\rm{out}}}\right\rangle },$$

we can calculate the equilibrium *e*^{0}^ of the dynamical system:

4 $$\frac{dx}{dt}=f(x)+{w}_{{\rm{av}}}g(x,x).$$

The method to derive this ODE is explained in the SI. The relative merits of the two way to define *w*_av_ are also discussed in SI, both are used frequently in literature^10^.

If *g*(*x*, *y*) = (*x* − *y*)*h*(*x*, *y*) and *f*(*e*) = 0, then the system has uniform equilibria in the form of *e***1**, where *e* is such that *f*(*e*) = 0. Let *L* be the in-Laplacian matrix of the graph and *λ*_*i*_ be its eigenvalues. If *h*(*e*, *e*) = *α* ≠ 0, then the Jacobian matrix at *e***1** is

$${J}_{e}={f}^{{\prime} }(e){{\rm{Id}}}_{N}+\alpha \ L,$$

where Id_*N*_ is the *N* × *N* identity matrix. For the eigenvalues, *μ*_*i*_, of *J*_*e*_ it holds that

$${\mu }_{i}=\alpha \ {\lambda }_{i}+{f}^{{\prime} }(e).$$

This gives a direct indication of system stability (*μ*_*i*_ < 0) as a function of the network topology (*λ*_*i*_) and the individual dynamics (${f}^{{\prime} }(e)$).

In the general case where the system does not have uniform equilibria, then we proceed to Step 1.

**Step 1**: We use the mean field approximation as an initial guess to bootstrap our approximations. We approximate the dynamics on each node by the dynamical system:

5 $$\frac{dx}{dt}=f(x)+{w}_{i}g(x,{e}^{\{0\}})=0.$$

See the SI for an explanation of how we get this equation. The solution of this equation is a function of *w*_*i*_, i.e. *χ*^{1}^(*w*_*i*_). Then our first order approximation is *e*^{0}^1_*i*_ = *χ*^{1}^(*w*_*i*_).

**Step 2**: We can use the previous approximation to approximate the effect that the graph has on a single vertex. Given a vertex *i* an effect an in-edge will have on the dynamics is *g*(*x*_*i*_, *x*_*j*_). In order to find the average effect of an in-edge, we have to notice that the probability of a vertex *j* is on the other side of the in-edge is proportional to its out-degree. With this in mind we can average over all possibilities and we find that the average effect is ${\sum }_{j=1}^{N}{d}_{j}^{{\rm{out}}}g({x}_{i},{x}_{j})/{\sum }_{j=1}^{N}{d}_{j}^{{\rm{out}}}$. In order to find their mean effect of the neighbours, each component of the coupling vector *g*(⋅) is weighted by *d*^out^. This means that we can use the previous step’s approximation and we find that the the equilibrium of the system can be approximated by the equilibrium of

6 $$\frac{dx}{dt}=f(x)+{w}_{i}\frac{{\sum }_{j=1}^{N}{d}_{j}^{{\rm{out}}}g({x}_{i},{{\bf{e}}}_{j}^{\{1\}})}{{\sum }_{j=1}^{N}{d}_{j}^{{\rm{out}}}}.$$

The solution of this equation depends on *w*_*i*_, i.e. *χ*^{2}^(*w*_*i*_). Then our second order approximation is ${e}_{i}^{\{2\}}={\chi }^{\{2\}}({w}_{i})$.

We define the slope at each vertex.

$${s}_{i}^{\{2\}}={f}^{{\prime} }\left(\right.{e}_{i}^{\{2\}}\left)\right.+{\sum }_{j=1}^{N}\left|{a}_{ji}\ {g}^{(0,1)}\left(\right.{e}_{i}^{\{2\}},{e}_{j}^{\{2\}}\left)\right.\right|.$$

If our approximation was exact that would be an upper bound for the eigenvalues of the Jacobian. However, since we have an approximation, if all ${s}_{i}^{\{2\}}$’s are negative and smaller than *F*(*e*^{2}^) when compared components-wise, this is a strong indication that the equilibrium is stable.

We define ${\mathcal{X}}=\{{\chi }^{\{n\}}({w}_{1}),\ldots ,{\chi }^{\{n\}}({w}_{N})\}$ and we define *e*^{0}^*n*_max_ to be the maximum possible value of **e**^{1}^*n*, i.e.

$${e}^{\{0\}}{n}_{{\rm{\max }}}={\max }_{w\in {\mathcal{W}},e\in {\mathcal{X}}}\max \{r\in {\mathbb{R}}:f(r)+w\ g(r,e)=0\}.$$

Similarly we define ${e}_{\,{\rm{\min }}\,}^{\{n\}}$ to be the minimum possible value of **e**^{*n*}^. These upper and lower bounds can indicate when the system has potentially lost resilience. This is discussed in results.

**Step 3 to n**: We repeat the above, using each time the approximation we calculated in the previous step.

Using the bounds we can find the region where a bifurcation can happen and the system can lose resilience. We find that outside of this region, as long as the assumptions are satisfied, our method works with good accuracy. However, there is no practical algorithm that can precisely bound the error of our approximation, i.e. every algorithm that does this has complexity at least equal to the complexity of solving the full system numerically. Instead we can gauge the error of our approximation by evaluating the dynamics on the approximation, *F*(*e*^{*n*}^). Since the vector field vanishes on the equilibrium, the better *e*^{*n*}^ approximates the equilibrium, the smaller the vector *F*(*e*^{*n*}^) becomes.

It is worth noting that even though our method shares some similarities with the Master Stability Function^8^, there are a couple of significant differences that prevent the application of the latter. Firstly we assume that the equation $\dot{x}=f(x)$ has an equilibrium and secondly in our case the interaction term ${\sum }_{j}^{N}{a}_{ji}g({x}_{i},{x}_{j})$ does not vanish when *x*_*i*_ = *x*_*j*_ for every *j*.

### Algorithm complexity

Every step of our method requires *O*(*N*) operations. The only exception is the computation of bound that require *O*(*N*^2^) operation in the worst case, but required only *O*(*N*) operations for the examples considered here. If we want to solve the system numerically we need (*N*^*ω*^), where *ω* usually has value 3 or 2.807. For more information see Section I-G of the SI.

### Resilience estimation

The resulting framework is a robust and accurate way of measuring the networked dynamics and resilience function at each node, with the ability to identify vulnerable nodes. We can generally predict the resilience function with up to 98% accuracy after *s* ≥ 2 steps of estimation. Furthermore, it mathematically links topological measures and non-linear dynamics (relationship shown in Fig. 1i-b). We demonstrate its capability through commonly studied ecological systems, subject to the standard perturbation models of: (i) node loss, (ii) link loss, and (iii) weight loss. We expect this new and transformative framework will map to existing application domain knowledge and inform the design and operations in a wide range of domains. The resulting framework is a robust and accurate way of measuring the networked dynamics and resilience function at each node, with the ability to identify vulnerable nodes. We can generally predict the resilience function with up to 98% accuracy after *s* ≥ 2 steps of estimation. Furthermore, it mathematically links topological measures and non-linear dynamics (relationship shown in Fig. 1). We demonstrate its capability through commonly studied ecological systems, subject to the standard perturbation models of: (i) node loss, (ii) link loss, and (iii) weight loss. We expect this new and transformative framework will map to existing application domain knowledge and inform the design and operations in a wide range of domains.

## Results

### Node level resilience

The key benefit of our proposed framework is the ability to identify vulnerable nodes that are at risk of losing resilience. This is done so by examining the impact of perturbations on the effective resilience of the whole network via the mean field approach^10^, and then sequentially inferring the node level impact.

**Figure 2:**
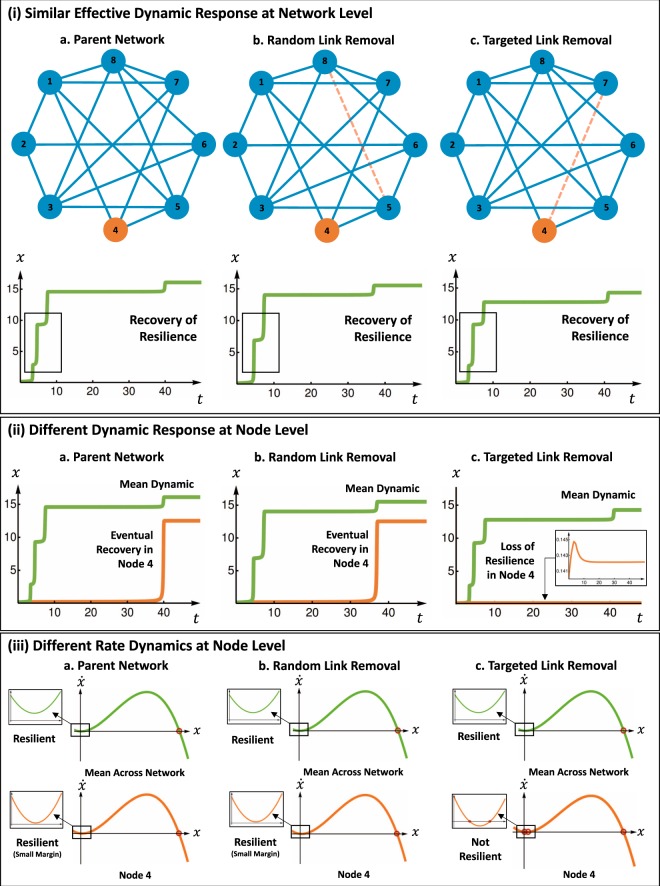
Similar Network Dynamics Hide Different Node Dynamics. (**i**) Similar Network Level Dynamic - (a) the parent network can have a link removed either (b) randomly or (c) targeted to cause local resilience loss at the node level. At the network level, the effective behaviour^10^ is similar: demonstrating that the whole system’s mean behaviour can recover. (**ii**) Different Node Level Dynamic - However, we show that there is a loss of resilience in node 4 for case (c), but not for case (a,b). Whilst this detail is lost in the network level mean behaviour, it can be predicted using our proposed framework. (**iii**) Different Node Level Rate Dynamics - This shows that whilst we retain a similar resilient profile across the network across all cases, we can clearly see that node 4 is marginally above resilience in the parent network, remains resilient after random link removal, but looses resilience after targeted link removal.

Here, our results in Fig. 2i show that a parent network (case a) can have a similar network-level effective dynamics after perturbation. In this example we consider both a random link removal (case b) and a targeted link removal (case c). We see that the network’s mean dynamic response is similar (small differences highlighted in black box). However, when we look at an individual node’s dynamic response (node 4 in Fig. 2ii), we observe 2 different effects. First, we see that node 4 recovers its desirable functionality (case a and b) with a longer delay. Second, we see that when targeted link removal (case c) is performed, node 4 never recovers (zoom in shows it collapses to a low equilibrium value).

These results highlight a shortfall in current approaches that only estimate network-level effective dynamics^10–12,15^, whereby all 3 cases have near identical mean field values and as such yield similar network-level dynamics. That means practitioners are unable to identify vulnerabilities at the node level and gain more insight or direct interventions.

Examining the node level results, Fig. 2iii shows the rate dynamics. Again, a similar network level behaviour exists before and after perturbations. However, at the node 4 level, we can see how targeted link removal can shift it from resilient with a small margin to not resilient. The dynamics used in Fig. 2 are: $f(x)={x}_{i}(1-\frac{{x}_{i}}{5})({x}_{i}-1)$, and $g({x}_{i},{x}_{j})=\frac{{x}_{i}^{2}}{2({x}_{j}+1)}$, and the estimation steps used is *s* = 1 (2 steps). Later in the paper, we will present more complex dynamical systems, where the results are less intuitive. For now, to motivate readers, we present 2 applied case studies motivated by examples given in^9,10^.

### Case study: ecological network

**Figure 3:**
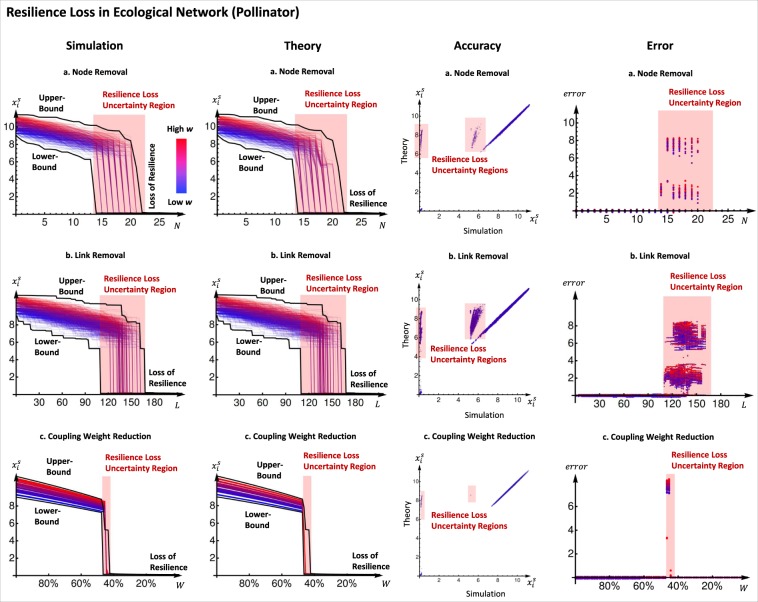
Resilience Function in Ecological Network (Pollinator). We disrupt the network by making it less connected in three different ways: (**a**) The first row shows the results for random node removal, 20 trajectories are shown. Numerical simulation of the equilibrium is shown in the first figure and 3-step approximation with our method is shown in second. In both figures the theoretical bounds are plotted in black. The third figure shows the simulation plotted against the approximation and the last figure shows the absolute error of the two. (**b**) The second row shows the results for random link removal, 20 trajectories are shown. Similarly to the previous case, numerical simulation of the equilibrium is shown in the first figure and 3-step approximation with our method is shown in second, with the bounds in black. The third and the fourth figures show the simulation plotted against the approximation and the absolute error of the two respectively. (**c**) The third row shows the results for uniform coupling weight loss. The same information as before is shown in the figures. In all three cases we see that the bounds predict the region where the bifurcation happens and the error is very small outside this region.

In Fig. 3, we use a well studied case of pollinator networks^16^. The abundance of species *i*, *x*_*i*_ is given by:

7 $$\frac{d{x}_{i}}{dt}={B}_{i}+{x}_{i}(1-\frac{{x}_{i}}{K})(\frac{{x}_{i}}{C}-1)+\mathop{\sum }\limits_{j}^{N}{a}_{ji}\frac{{x}_{i}{x}_{j}}{{D}_{i}+{E}_{i}{x}_{i}+{H}_{j}{x}_{j}},$$

where with reference to Eq. 2, *f*(.) is a logistic growth equation balancing the carrying capacity *K*_*i*_ with the Allee effect (low abundance *x*_*i*_ < *C*_*i*_ leads to population decline), *g*(.) in Eq. 2 is a coupling function with saturation, and *B*_*i*_ is a constant migration rate from other ecosystems. For simplicity, we use homogeneous parameters: *B* = 0.1, *C* = 1, *K* = 5, *D* = 5, *E* = 0.9, *H* = 0.1. For topological generality in all our case studies, we used random graphs and in this case it is a Erdős-Rényi graph with *N* = 30 nodes and a connectivity factor of *p* = 0.5. Other random graphs exhibit similar results and are not presented here. In this case study, we define system resilience by the ability of the system to recover all the populations after extinction. This is possible because we assume that seeds can come to the geographic location from nearby locations, where there are still healthy plants. This is the reason we define the parameter *B* to be non-zero. However, in order for this to happen, the system has be in a region of the parameter space where there is one stable equilibrium. If there are two stable equilibria, the system will be trapped in the one with low population density, thus cannot recover. Similarly we can also define node resilience by the ability of a species to recover its population after near extinction. The specific dynamics used here imply that as long as a node is connected to another healthy one, it is resilient, see II-A1 in SI for an explanation why this is the case.

In Fig. 3 we show what happens when a network become less connected in three different ways: (i) by removing nodes, (ii) by removing edges and (iii) by reducing globally the weight. The results shown are 20 random trajectories for one Erdös-Rényi graph. In the SI we give similar results for Barabasi-Albert and Watts-Strogatz graphs.

The results show that outside the resilience loss regime (red region), we are able to predict well the effect of the different perturbations, with less than 2–4% error with estimation steps *s* = 3 and an initial mean field approximation of *w*_av_. From the results, we can see that due to the Allee effect, the collapse in abundance in every species is dramatic after a certain perturbation level. We are able to create upper- and lower-bounds for the dynamics, such that we can estimate the size of the uncertainty region. We can see that within the uncertainty region, the error can be arbitrarily large - highlighting unpredictable behaviour during resilience loss. The impact of this work is that we can clearly predict the onset of resilience loss for different measurable perturbation dynamics. We can see the impact of changing either specific species parameters (e.g. carrying capacity or colony threshold) and overall spatial network level connectivity on the resilience profile of both the whole ecosystem and the specific species.

### Case study: biological network

**Figure 4:**
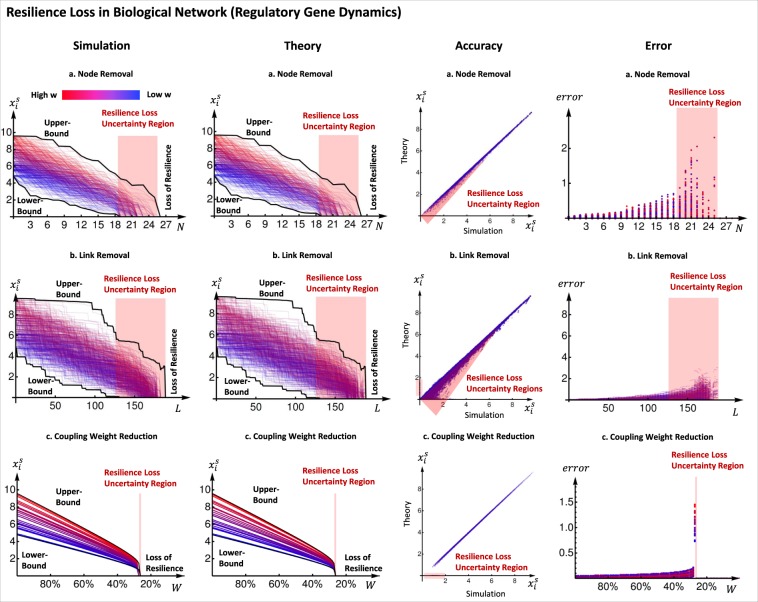
Resilience Function in Biological Network (Gene Regulation). Similarly to the Ecological Network case, We disrupt the network by making it less connected in the same three ways: (**a**) The first row shows the results for random node removal, 20 trajectories are shown. The first figure shows the numerical simulation of the equilibrium and the second the 1-step approximation with our method. In both figures the theoretical bounds are plotted in black. The simulation plotted against the approximation is shown in the third figure and the last figure shows the absolute error of the two. (**b**) The second row shows the results for random link removal, 20 trajectories are shown. Similarly to the previous case, numerical simulation of the equilibrium is shown in the first figure and 1-step approximation with our method is shown in second, with the bounds in black. The third and the fourth figures show the simulation plotted against the approximation and the absolute error of the two respectively. (**c**) The third row shows the results for uniform coupling weight loss. The same information as before is shown in the figures. Similarly to the Ecological Network case, in all three cases we see that the bounds predict the region where the bifurcation happens and the error is very small outside this region.

In Fig. 4, we show the corresponding results using a well studied case of gene regulatory networks governed by the Michaelis-Menten Eq.^17^, given by:

8 $$\frac{d{x}_{i}}{dt}=\,-\,{x}_{i}^{a}+{\sum }_{j}^{N}{a}_{ji}\frac{{x}_{j}^{h}}{2({x}_{j}^{h}+1)},$$

where with reference to Eq. 2, *f*(.) is a degradation (*a* = 1) or dimerization (*a* = 2) effect, and *g*(.) in Eq. 2 is genetic activation, where the Hill coefficient *h* describes the level of cooperation in gene regulation. Using *a* = 1, *h* = 2, we find that there is a more gradual loss of resilience than the pollinator network.

For this system resilience is defined differently. If activity in this system ceases, then the cells die and as a result there is no possibility of recovery. This is the reason that for the dynamical system the state where every variable is equal to 0 is always a stable equilibrium. This means that we define resilience to be the ability of the system to stay alive and recover from a mild slow down of activity. We say that the system loses resilience if it move to a region of the parameter space where 0 is the only stable equilibrium, since if this happens all activity halts.

We show that outside the resilience loss regime (red region), we are able to predict well the effect of perturbations, with an initial error of less than 2% (rising gradually), with estimation steps *s* = 0 (1 initial mean field step, because the dynamics are trivial) and the initial mean field approximation of $\frac{\langle {w}_{{\rm{in}}}{w}_{{\rm{out}}}\rangle }{\langle w\rangle }$ used in^10^. We are also able to create upper- and lower-bounds for the dynamics, such that we can estimate the size of the uncertainty region. This case study demonstrates that when the dynamics are relatively trivial (no *x*_*i*_ in coupling dynamics *g*(.)), we can predict the gradual resilience loss very well. Later in the next section, we will show how a critical resilience function can be used to identify the most vulnerable nodes and how for non-trivial cases, the resulting resilience functions can be non-intuitive.

### Resilience bounds and critical resilience value

**Figure 5:**
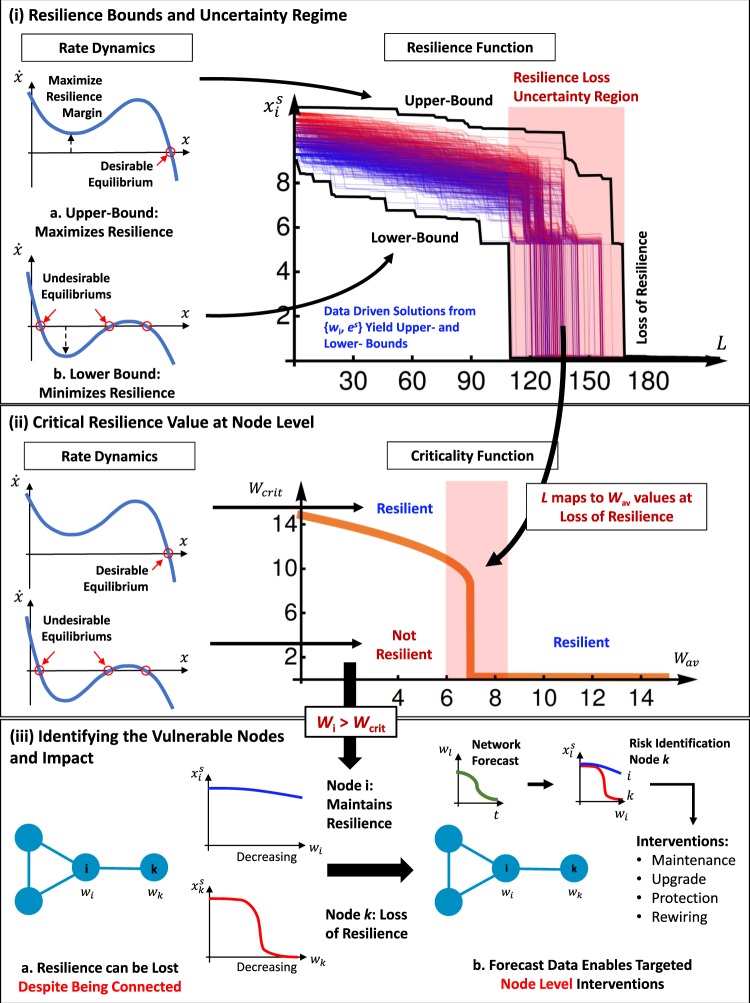
Critical Resilience Value Identifies Vulnerable Nodes. (**i**) Resilience Bounds - Bounds on resilience loss yield theoretical prediction envelope. The bounds explicitly map to average weighted degree values (*w*_av_), which map to the critical resilience value plot in (ii). Uncertainty in behaviour increases during critical collapse phase. (**ii**) **Criticality Function** defines resilience regimes mapping network properties (average weighted degree *w*_av_) to local node properties (critical resilience value *w*_crit_). When *w*_*i*_ > *w*_crit_, the node is resilient, and when below it is not. This is a way to identify which nodes are likely to be vulnerable to a loss of resilience. (**iii**) **Impact** - This shows that nodes do not have to be removed to lose resilience. By being able to identify and forecast which nodes are at risk of resilience loss as a function of parameters (e.g. declining interactions *w* over time), we can target interventions for different application contexts.

The crux of our work is to tractably analyze node level resilience and use this to identify which nodes are at risk of loosing resilience. In Fig. 5, we use the pollinator dynamics (see Eq. 7) to demonstrate how to estimate the upper- and lower-bounds of the resilience function and identify vulnerable nodes. All the resilience behaviour at each node (e.g. loss of performance metric *x* as a function of link removal *L*) is bounded by the theoretical bounds.

The bound solutions are subject to the real data available on both the topology (*w*_*i*_) and the equilibrium estimation *e*^*n*^. When the resilience bounds collapse, we can use their *w*_av_ values to map the uncertainty regime to other relationship plots. One such plot is the criticality function. Here, we define the critical resilience *w*_crit_ as the value by which each node must satisfy *w*_*i*_ > *w*_crit_ in order to stay resilient. This condition enables us to identify which nodes close to losing resilience - see Fig. 2iii, despite being reasonably well connected, and use future knowledge of connectivity changes to drive forecasting of resilience loss at the node level.

### Network rewiring and unwiring

An important notion for dynamical systems on graphs is the *critical weight* of a node. Given the dynamics, the critical weight is the minimum weighted degree a node needs in order to be resilient. This is a function of the average weight of the graph, see the SI for the exact derivation. In the case of pollinator dynamics, the higher the average weight is, the higher the mean field solution is. With high mean field solution a node can be resilient with low weighted degree. So we see that intuitively the critical weight is a non-decreasing function of the average weight.

However, this does not happen in every case. We now show that for the dynamics *f*(*x*_*i*_) = 1/10 + *x*_*i*_(1 − *x*_*i*_)(*x*_*i*_/5 − 1) and $g({x}_{i},{x}_{j})=15({x}_{i}^{2}{x}_{j})/(1+{x}_{j}^{2})$, changes to the coupling dynamics in one part of the network can cause counter-intuitive changes to the resilience in another part of the network. In this case, increasing the connectivity of one part of the network can have opposite effects on the resilience of other nodes, depending on their local connectivity and global network topology.

#### Intuitive rewiring to restore resilience

**Figure 6:**
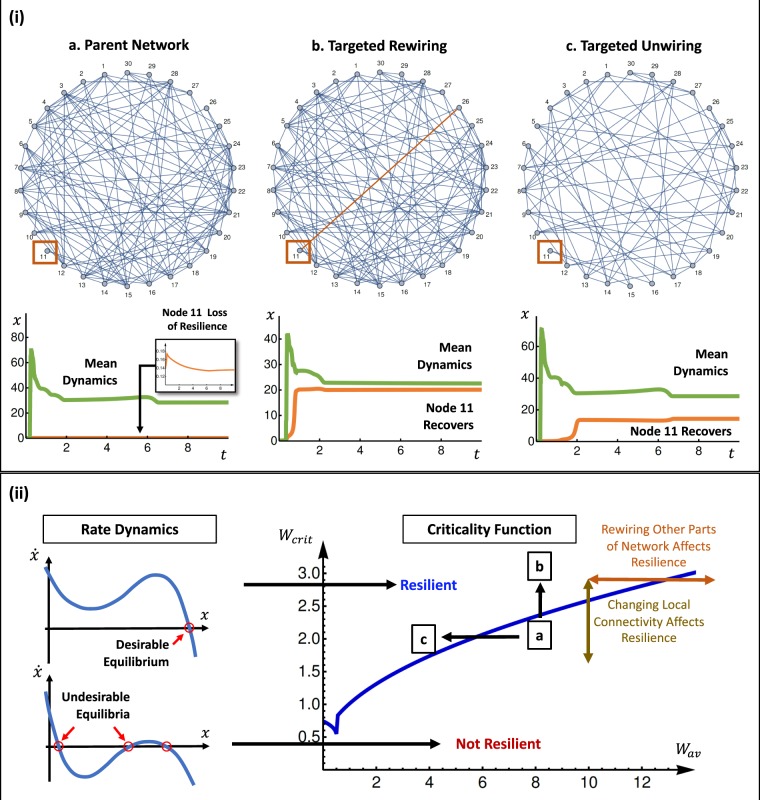
Network Rewiring & Unwiring to Change Resilience. (**i**) (a) parent network’s node 11 has failed, (b) targeted rewiring by adding an edge allows node 11 to recover, (c) targeted unwiring of other parts of the network allows node 11 to recover. (**ii**) criticality function with inflection point, showing that rewiring/unwiring one part of the network has different effects on the resilience for other parts of the network. In our example, the parent network’s node 11 moved from a not resilient regime (a) to a resilient regime by increasing its own connectivity (b) or unwiring other network parts (c).

In Fig. 6i, we consider a parent network (case a), whereby node 11 has already lost resilience - see dynamics *x*(*t*). In Fig. 6ii, the position of node 11 on the criticality function is labelled and is in a not resilient regime. When we perform targeted rewiring (case b), node 11 is connected to node 26 and its dynamic response recovers. This is intuitive, because rewiring improves its local weighted degree *w*_11_ > *w*_crit_ and shifts the position upwards into a resilient regime.

#### Intuitive unwiring to restore resilience

An alternative and non-intuitive way of restoring resilience to node 11 is to unwire a distant part of the network. When we perform targeted link removal (case c), this reduces the average weighted degree *w*_av_ such that it shifts its position of node 11 on the criticality function leftwards into a resilient regime (case c).

In summary, it is entirely plausible to have a system whereby its dynamics enables both intuitive (e.g. local rewiring boosts resilience) and counter-intuitive results (e.g. distant unwiring boosts resilience). In such cases, making a small change in one part of the network can dramatically improve resilience for some nodes, whilst reducing resilience for others, depending on where they are with respect to critical inflection point in Fig. 6ii. The fact that local changes can affect resilience in a completely different part of the network deserves attention and further research. Suffice to say, without a fundamental understanding of the criticality function through our proposed sequential heterogeneous mean field approach, we cannot determine the impact of wiring and unwiring at the node level.

## Discussion and Limitations

A gap in understanding exists between individual dynamics and the coupled dynamics in a large-scale networked complex system. Here, we present a framework for tractably analyzing the resilience of individual nodes as a function of the individual dynamics and the network property. We show that our method can be used to:

1. *Estimate the equilibrium behaviour outside the critical region*. Our methods gives an accurate approximation of the equilibrium when the system is not close to the critical region.
2. *Estimate the critical region as a function of the perturbation and identify which nodes are most vulnerable to loss of resilience*. The collapse of the lower bound indicates that there exist nodes that are about to lose resilience. The approximation correctly predicts the order in which the vertices lose resilience.
3. *Predict the effect of changing the network on the resilience of nodes*. As shown in Section II-E, we were able to predict a counter-intuitive behaviour of a system.

Whilst our baseline result is intuitive (e.g. the most vulnerable nodes are poorly connected ones close to the critical resilience value *w*_crit_), quantifying this value as a function of the dynamics enables us to prioritize actions more effectively and predict resilience loss more accurately. Conversely, we may also discover hidden cascade effects, whereby disconnecting a weakly connected node can lead to improvement in other nodes. This is useful for analyzing recent claims on eradicating the malaria mosquito because it is not a significant diet for predators (e.g. weak basal species^18^). This maybe risky, because we do not know the underlying dynamics nor the resilience margins in all connected species in the food web.

It is also useful to discuss when our estimation algorithm doesn’t work. As with^10^, the estimation produces increasing errors with (1) increasing degree correlation (assortiveness). This is due to the fact that the neighbours a given vertex has depends on its degree, which our approximation of the effect these neighbours have inaccurate. Putting this caveat aside, in general, the node-level precision methods developed here will enable practitioners in ecology, infrastructure, and other application areas to prioritize protection and intervention resources, such as maintenance, preservation, rewiring, and upgrades. Future work will extend this research to consider both local^19^ and global^20,21^ optimal control of complex network dynamics.

## Supplementary information

Supplementary Information.
