# Supplementary material for: Node-Level Resilience Loss in Dynamic Complex Networks
Source: Sci Rep. 2020 Feb 27;10:3599. doi: 10.1038/s41598-020-60501-9 (PMC7046645; doi:10.1038/s41598-020-60501-9)
Supplement: Supplementary file 1 — Supplementary Information. [file 41598_2020_60501_MOESM1_ESM.pdf]

# Node-Level Resilience Loss in Dynamic Complex Networks - Supplemenatary Information

Giannis Moutsinas, Weisi Guo

## I. Exposition of the Method

Let $\mathcal{G}$ be a weighted directed graph of $N$ vertices and $A$ be its weighted adjacency matrix. We associate each vertex $v_i$ with the function of time $x_i$, that we call the *state* of $v_i$ and we assume that $x_i$'s satisfy the dynamical system

$$\dot{x}_i = f(x_i) + \sum_{j=1}^{N} a_{ji} g(x_i, x_j), \tag{1}$$

where $f : \mathbb{R} \to \mathbb{R}$ and $g : \mathbb{R}^2 \to \mathbb{R}$, $a_{ji} = (A)_{ji}$. Our goal is to describe the equilibria of this system and their stability. We start by finding the mean field approximation of the system and we iteratively refine this prediction.

We make the following assumptions:

1) The functions $f$ and $g$ are twice differentiable and there exists $R \in \mathbb{R}$ such that for all for all $|x|$, $|y| > R$ and $w \in \mathbb{R}^+$, $(f(x) + wg(x, y))\, x < 0$.
2) The weights are independent and identically distributed random variables, with positive mean.
3) The graph has low degree correlation.

*Assumption 1:* This assumption implies that the state of the system cannot escape to infinity. For a given system we may be able to relax this a bit by assuming that it holds for $w$ in a given interval. In Section I-C we discuss briefly an example of how our method can fail if the first assumption is not satisfied.

*Assumption 2:* The second assumption implies that the in-degree and the weighted in-degree of a vertex are correlated, i.e. the weighted degree of a vertex can be used as an indicator of how connected the vertex is. The smaller the mean is in comparison to the standard deviation, the less correlated the two degrees become. This increases the error of our approximation.

The assumption allows us to use the weighted in-degree instead of the in-degree of a vector when we calculate the average effect of the neighbourhood. On the extreme case that the standard deviation of the weights is $0$ then the weighted in-degree is a multiple of the in-degree and this substitution does not produce any error. On the other hand, if the average weight is $0$, then the two degrees are uncorrelated. This means that a vertex can have high in-degree and at the same time weighted in-degree $0$. In this case our approximation will find that the neighbours have not effect, whereas the neighbours' effect can be significant. Between these two extremes lies a continuum of possibilities. We find that when the standard deviation is of similar size to the mean, our approximation works well, as it is with the cases we discuss in the paper.

*Assumption 3:* The last assumption implies the network is not assortative in any type of degree assortativity and that the neighbourhoods of the vertices are in a sense uniform. This is an important assumption that allow us to approximate the effect that the neighbourhood has on the vertex by the effect of the"average neighbourhood". Note that assumption 2 allows us to use the weighted in-degree to calculate the "average neighbourhood" of a vertex. In particular this assumption implies that given a vertex $i$, the probability of a vertex $j$ being an in-neighbour of $i$ is proportional to the out-degree of $j$ and independent to the in-degree of $i$.

We find that for graphs with high assortativity our method has a significant error, for example spatial graphs. On the other hand for graphs that are constructed using the WattsStrogatz model that have high clustering coefficient but low assortativity, we find that our method has very good accuracy.

### A. Notation

Let $w_i$ be the *weighted in-degree* of vertex $v_i$, i.e.

$$w_i = \sum_{j=1}^{N} a_{ji},$$

and we denote by $w_{\text{av}}$ the (potentially weighted) average of all weighted in-degrees. We denote by $w_j^{\text{out}}$ the *weighted out-degree* of vertex $v_j$, i.e.

$$w_j^{\text{out}} = \sum_{i=1}^{N} a_{ji}.$$

Similarly we denote by $d_i$ the in-degree of $v_i$ and by $d_i^{\text{out}}$ its out-degree. We define the closed interval $\mathcal{W}_{\mathcal{G}} = [\min\{w_i\}, \max\{w_i\}]$ and lastly for a vertex, $v$, we denote by $\mathcal{N}(v)$ its neighbourhood.

We denote by $\mathbf{1}$ the $N$-vector, whose entries are all 1. We will call $X \in \mathbb{R}^N$ a uniform vector if there exists $x \in \mathbb{R}$ such that $X = x\,\mathbf{1}$. Similarly, if an equilibrium is a uniform vector, we will call it a uniform equilibrium. Let $X^T = (x_1, \ldots, x_N)$, then we can rewrite the system (1) in the form

$$\dot{X} = F(X), \tag{2}$$

with $F : \mathbb{R}^N \to \mathbb{R}^N$ defined by equation (1). We define $\mathcal{F} : \mathbb{R}^N \to \mathbb{R}$ by $\mathcal{F}(X) = E[F(X)]$.

Note that we have not specified yet how exactly this mean is calculated. We will present two possibilities here, the uniform mean and the out-degree weighted mean. The uniform mean counts every component of the vector $F(X)$ with the same weight and it is the most intuitive one. The out-degree weighted mean we weight the $i$-th component of the vector $F(X)$ with $w_i^{\text{out}}$. This is less intuitive but it captures the fact that given a vertex $v_i$ the probability that a vertex $v_j$ is its neighbour is proportional $w_j^{\text{out}}$, see Section I-D for a brief explanation or [1] for an in depth discussion. We will denote this mean by $E_{\text{out}}$ In the latter case the mean field approximation is the same as in [2].

### *B. Mean Field Approximation*

For the mean field approximation, we try to approximate the equilibrium of the system by a uniform vector. If the equilibrium is uniform, then there exists $e \in \mathbb{R}$ such that $F(e\mathbf{1}) = 0$. Of course this is not true in general, so we are looking for $e \in \mathbb{R}$ such that $\mathcal{F}(e\mathbf{1}) = 0$.

As we noted in the previous section there are two somewhat obvious choices for the mean. We find that the uniform mean tends to underestimate value of the equilibrium and the out-degree weight mean tends to overestimate it. However when we used the latter, the next approximations have the same or sometimes better accuracy than when we used the former. Nevertheless, the uniform mean may still be proven useful, especially as a second guess about the dynamics. This opens up the possibility of defining a continuum of ways to take the mean using as weights $(d^{\text{out}})^\alpha$ with $\alpha \in [0, 1]$. We defer the discussion of finding the optimal value for $\alpha$ as a function of the topological properties of the graph to future work.

We will be using the out-degree weighted average, so $w_{\text{av}} = E_{\text{out}}[w_i]$. This implies that

$$\begin{aligned} \mathcal{F}(e\,\mathbf{1}) &= E_{\text{out}}[F(e\mathbf{1})] \\ &= f(e) + E_{\text{out}}[w_i]\; g(e, e). \end{aligned} \tag{3}$$

This implies that every $e$ that satisfies equation (3) potentially approximates an equilibrium of the system.

If $e\mathbf{1}$ approximates well the equilibrium, we can look at the stability of the "average vertex" by assuming that the state of every vertex is fixed at $e$. More precisely we look at the *slope*

$$s = f'(e) + w_{\text{av}}\, |g^{(1,0)}(e,e)|.$$

If $s > 0$ the equilibrium that $e$ approximates is unstable. However the condition $s < 0$ is not sufficient for stability of the system.

We will call the mean field approximation the *zeroth order approximation of the equilibrium*. From here on, we will denote the number $e$ by $e^{\{0\}}$, vector $e^{\{0\}}\mathbf{1}$ by $\mathbf{e}^{\{0\}}$ and the number $s$ by $s^{\{0\}}$

*1) Systems with Uniform Equilibria:* We will briefly discuss the class of systems for which the mean field approximation is exact. These are systems where $g(x,y) = (x-y)h(x,y)$. For such systems if $f(e) = 0$, then $F(e\mathbf{1}) = 0$.

Let $D$ be the diagonal matrix with $(D)_{ii} = w_i$ for all $i$, $L = D - A$ be the Laplacian matrix of the graph and $\lambda_i$ be its eigenvalues. Then the Jacobian matrix at $e\mathbf{1}$ is

$$J_e = f'(e)\text{Id}_N + H(e,e)\, L,$$

where $\text{Id}_N$ is the $N \times N$ identity matrix. For the eigenvalues, $\mu_i$, of $J_e$ it holds that $\mu_i = h(e,e)\,\lambda_i + f'(e)$.

### C. First Order Approximation

Once we have the mean field approximation, we can look at the dynamics of a single vertex. We choose a vertex $v_i$ and we assume that the state of all its neighbours is $e^{\{0\}}$ and that the state of $v_i$ does not affect them. Under these assumptions the dynamics of $v_i$ is given by

$$\dot{x}_i = f(x_i) + \sum_{j=1}^{N} a_{ji} g\big(x_i, e^{\{0\}}\big) = f(x_i) + w_i\, g\big(x_i, e^{\{0\}}\big). \tag{4}$$

The equilibrium of the above dynamics will approximate the value of the equilibrium for $v_i$. Let us define $g_{\{1\}}(x) = g\big(x, e^{\{0\}}\big)$ and let $\chi^{\{1\}}(w)$ denote the root of the function

$$G_{\{1\}}(w,x) = f(x) + w\, g_{\{1\}}(x) \tag{5}$$

that is closer to $e^{\{0\}}$. If $\chi^{\{1\}}$ is not defined everywhere on $\mathcal{W}$ or is discontinuous, then this is an indication that the approximation is not accurate and the equilibrium we try to approximate might not exist.

If the function $\chi^{\{1\}}$ is continuous on $\mathcal{W}$, then we can update our prediction about the state of vertex $v_i$ from $e^{\{0\}}$ to $e_i^{\{1\}} = \chi^{\{1\}}(w_i)$. We define

$$\mathbf{e}^{\{1\}} = \{e_1^{\{1\}}, \dots, e_N^{\{1\}}\}$$

and we call this the *first order approximation of the equilibrium.* One way to guess the order of our approximation by looking at the values of $F(\mathbf{e}^{\{1\}})$.

Similarly to the mean field approximation, in order to determine the stability of the system, we define

$$s_i^{\{1\}} = f'\big(e_i^{\{1\}}\big) + \sum_{j=1}^{N} |a_{ji}\, g^{(1,0)}\big(e_i^{\{1\}}, e_j^{\{1\}}\big)|$$

Since $\mathbf{e}^{\{1\}}$ approximates the equilibrium, $F'(\mathbf{e}^{\{1\}})$ approximates the Jacobian. Then by the Gershgorin circle theorem [3] we get that $\max s_i^{\{1\}}$ is an upper bound for the real part of the eigenvalues of the Jacobian evaluated at the approximation and an approximate upper bound of the eigenvalues of the equilibrium.

The vector $F(e^{\{1\}})$ gives the the vector field at the point $e^{\{1\}}$, if all $s_i^{\{1\}}$'s are negative and $F(e^{\{1\}})$ is component-wise smaller in absolute values, it is a good indicator that the equilibrium is stable.

*Unboundedness of solutions*

It is interesting at this point to discuss briefly the the case where Assumption 1 does not hold. As an example let us use

$$f(x) = \frac{1}{10} - 5x + x^2,$$
$$g(x, y) = x\, y.$$

We see that function $f$ has an unstable equilibrium close to $5$ and if a trajectory is on the right of this, then it escapes to infinity.

This means that we may have the case where the mean field approximation of the system

$$\dot{x} = \frac{1}{10} - 5x + x^2 + w_{\text{av}} x^2$$

has a stable equilibrium. However, when we look at the vertex level dynamics

$$\dot{x}_i = \frac{1}{10} - 5x + x^2 + w_i e^{\{0\}} x_i$$

of a node with high $w_i$ we may see that there is actually no equilibrium at all and that the trajectory

escapes to infinity. This will have a domino effect on the whole graph and every vertex will escape to infinity. This means that in such system there exists the possibility of a catastrophic failure that could in principle be undetectable, thus extra care is required before drawing any conclusions.

### *D. Average Effect of the Neighbours*

For the first order approximation we looked at a vertex and we assumed that all its neighbours have the same state. Now that we have a better guess about the state of each vertex, we can update our guess about the states of the neighbours.

We pick a vertex $v_i$ and an in-edge of that vertex and we ask what is the probability that a given vertex $v_j$ is on the other end of the edge. This question is not solved in general for any distribution of random graphs. However, in the configuration model for random graphs, the probability for $v_j$ is approximately weighted by its out-degree $d_j^{\text{out}}$. The error of this approximation for large graphs constructed by the configuration model is of the order of $\frac{1}{N}$. Under our assumptions it is a reasonable approximation for the graph $\mathcal{G}$.

Let $q_i$ be a scalar quantity associated with vertex $v_i$ and $Q = \{q_1, \dots, q_N\}$, since the out-degree and the weighted out degree of a node are correlated, we define the weighted average

$$E_{\text{out}}[Q] = \frac{\sum_{j=1}^{N} w_j q_j}{\sum_{j=1}^{N} w_j}.$$

We can define higher moments the usual way, for example the variance is defined by

$$\text{var}_w[Q] = E_{\text{out}}[Q^2] - E_{\text{out}}[Q]^2,$$

with $Q^2 = \{q_1^2, \dots, q_N^2\}$. For any $\Phi : \mathbb{R} \to \mathbb{R}$, we denote by $\Phi(Q)$ the vector $\{\Phi(q_1), \dots, \Phi(q_N)\}$, then we have

$$E_{\text{out}}[\Phi(Q)] = \frac{\sum_{j=1}^{N} w_j \Phi(q_j)}{\sum_{j=1}^{N} w_j}.$$

### *E. Second and Higher Order Approximations*

It is clear that we can continue the above process indefinitely. Let us assume that we know the $n$-th order approximation of the equilibrium, $\mathbf{e}^{\{n\}}$. Then just like before we define the function $g_{\{n+1\}}(x) =$

$E_{\text{out}}\big[g\big(x, \mathbf{e}^{\{n\}}\big)\big]$ and the function

$$G_{\{n+1\}}(w, x) = f(x) + w\, g_{\{n+1\}}(x).$$

We find the roots of $G_{\{n+1\}}$ to get the function $\chi^{\{n+1\}}$ and define $e_i^{\{n+1\}} = \chi^{\{n+1\}}(w_i)$ and $\mathbf{e}^{\{n+1\}} = \{e_1^{\{n+1\}}, \ldots, e_N^{\{n+1\}}\}$.

We can assess how well each step approximates the equilibrium by looking at the vector $F\big(\mathbf{e}^{\{n\}}\big)$. We know that if $\mathbf{e}_*$ is an equilibrium, then $F\big(\mathbf{e}_*\big) = 0$. This means that the closer $F\big(\mathbf{e}^{\{n\}}\big)$ is to $0$, the better $\mathbf{e}^{\{n\}}$ approximates $e_*$. Then at each step we calculate $\big\|F\big(\mathbf{e}^{\{n\}}\big)\big\|$ and compare with the previous. We choose to stop when the improvement becomes marginal.

Since we can continue this process indefinitely, we can define a sequence of approximations $\{\mathbf{e}^{\{n\}}\}_{n\geq 0}$. Even though each approximation seems to be better than the previous and the sequence appears to converge to a limit in all examples we tried, we do not currently know whether this is actually the case and we will make no claim in this direction.

In order to determine the stability of the system, we define

$$s_i^{\{n+1\}} = s_i^{\{n+1\}} + \sum_{j=1}^{N} \Big| a_{ji}\, g^{(0,1)}\big(e_i^{\{n+1\}}, e_j^{\{n+1\}}\big)\Big|$$

Similarly to what we had in the case of the first order approximation, $\max s_i^{\{n+1\}}$ is an upper bound for the real part of the eigenvalues of the Jacobian evaluated at the approximation and an approximate upper bound of the eigenvalues of the equilibrium.

Moreover we compare component-wise the vector $F(\mathbf{e}^{\{n+1\}})$ with the slopes. If the slopes are negative and are equal or smaller than the absolute values of the components of $F(\mathbf{e}^{\{n+1\}})$, then we can be certain that the equilibrium is stable.

### *F. Upper and Lower Bounds*

Let $W = \{w_1, \ldots, w_N\}$ and $\mathcal{X} = \{\chi^{\{n\}}(w_1), \ldots, \chi^{\{n\}}(w_N)\}$. Then we define $e_{\text{max}}^{\{n\}}$ to be the maximum possible value of $\mathbf{e}^{\{n\}}$, i.e.

$$e_{\text{max}}^{\{n\}} = \max_{w\in\mathcal{W}, e\in\mathcal{X}} \max\{r \in \mathbb{R} : f(r) + w\, g(r, e) = 0\}.$$

Similarly we define $e_{\text{min}}^{\{n\}}$ to be the minimum possible value of $\mathbf{e}^{\{n\}}$.

The bounds are not very useful when the system is away from a bifurcation point, because the approximations tend to be quite accurate. However it can be used to detect the range in which the bifurcation may happen. We will see their usage in two examples: mutualistic interactions in ecological networks and gene regulatory networks.

### *G. Assessing the accuracy of the approximation*

Our approximation may not be accurate if the system is close to a bifurcation point. By using the bounds we can detect that. If the upper bound gives a resilient state and the lower bound gives a non-resilient state, then we know that the system is close to a bifurcation point.

Outside this region, the approximation is close to the equilibrium. Since for the equilibrium $\mathbf{e}_*$ we have $F(\mathbf{e}_*) = 0$, the better $e^{\{n\}}$ approximates $e^*$, the smaller $|F(e^{\{n\}}|$ becomes. So we can use the norm of the vector $F(e^{\{n\}})$, to gauge the error of our approximation.

### *H. Complexity of the Algorithm*

For fixed precision and fixed $f$ and $g$, the complexity of scalar operations and function evaluations is constant, i.e. $O(1)$. We are interested to see how the complexity depends on the number of vertices in the graph, $N$.

The complexity of the multiplication of 2 $N \times N$ matrices is $O(N^\omega)$, with $\omega = 3$ if we use the schoolbook matrix multiplication. The fastest algorithm that is used in practical applications is the Strassen algorithm and has $\omega = 2.807$. The fastest algorithm asymptotically is the optimised CoppersmithWinograd algorithm, with $\omega = 2.373$. However the constants involved in the complexity of this algorithm are big and in most practical applications the Strassen algorithm is faster.

Matrix inversion and solving a linear system have the same complexity as matrix multiplication, $O(N^\omega)$. This means that the Newton method for a function $F : \mathbb{R}^N \to \mathbb{R}^N$ for fixed precision has the same complexity. The power iteration method for calculating the largest eigenvalues also has the same complexity. Thus the complexity of calculating the equilibrium of the system and its stability is $O(N^\omega)$.

For the method we propose the overall complexity is $O(N)$. In the case of the mean field approximation we need to compute $w_{\text{av}}$, that has complexity $O(N)$ and then to solve a scalar equation, $O(1)$. Then for ever subsequent step we need to solve $N$ scalar equation, thus the complexity is again $O(N)$. The computation of $S^{\{n\}}$ also requires $O(N)$ operations. The computation of the bounds requires to check $O(N^2)$ pairs in the worst case. However since the functions are twice differentiable in many case we can easily

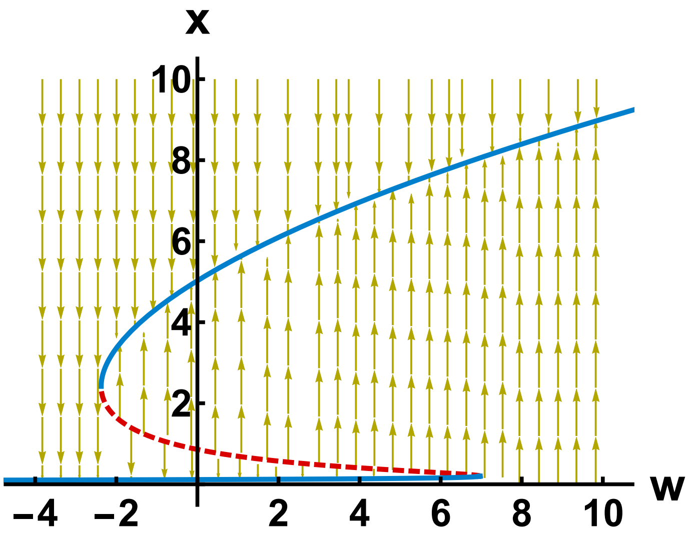


Fig. 1: The bifurcation diagram of (6). The stable equilibria are marked with a solid blue line and the unstable equilibrium is marked with a dashed red line.

guess the correct pair. For example in the cases considered here it is easy to see that maximum pair is $(\max_i(w_i), \max_i(e_i^{\{n\}}))$, similarly the minimum is $(\min_i(w_i), \min_i(e_i^{\{n\}}))$. This requires $O(N)$ operations.

## II. Applications

Similarly to [2], we will apply our technique in the case of mutualistic interactions in ecological networks and in gene regulatory networks. We have tried our method using graphs created by different models. We found that it works equally well in the case of Erdős-Rényi, Barabási-Albert and Watts-Strogatz models. We decide to use the Erdős-Rényi model in our exposition.

### A. *Mutualistic Interactions in Ecological Networks*

The dynamics in this model is given by

$$f(x) = B + x\left(\frac{x}{K}\right)\left(1 - \frac{x}{C}\right),$$
$$g(x,y) = \frac{x\,y}{D + E\,x + H\,y}$$

We follow [2] and we set $B = 0.1$, $C = 1$, $D = 5$, $E = 0.9$, $H = 0.1$ and $K = 5$. As we saw earlier, the 1-dimensional dynamics

$$\dot{x} = B + x\left(\frac{x}{K}\right)\left(1 - \frac{x}{C}\right) + w\,\frac{x^2}{D + (E+H)x} \tag{6}$$

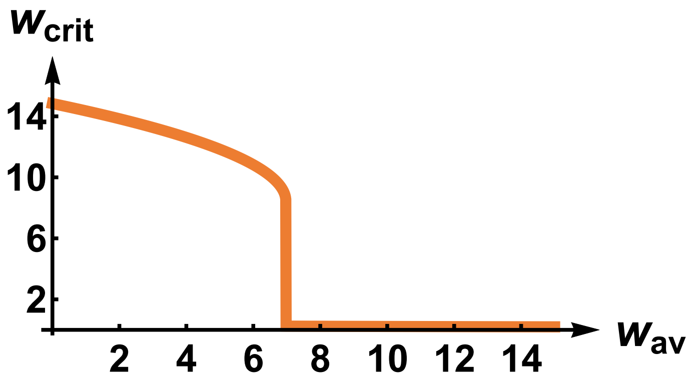


Fig. 2: The critical weight for mutualistic interactions dynamics.

serves as the mean field approximation. In this case, as the parameter $w$ varies the system undergoes two saddle-node bifurcations, see [4]. The first bifurcation happens at $w_*^- \approx -2.37$ and the second at $w_*^+ \approx 6.97$. The bifurcation diagram of the dynamical system (6) is shown in Figure 1. For $w < w_*^-$ the system has only one stable equilibrium $x_*^0(w)$ that is close to 0, $0 < x_*^0(w) < 0.3$. Similarly when $w > w_*^+$ the system has only one stable equilibrium $x_*^+(w)$ that does not approach 0, $x_*^+(w) > 2.3$. In the case when $w_*^- < w < w_*^+$, the system has both the above mentioned stable equilibria and between them another unstable one. Since we model mutualistic interactions, we do not have a physical interpretation of the case $w < 0$.

In this case $x$ describes the population of a plant species. The stable equilibrium $x_*^0(w)$ corresponds to the extinction of the species and we consider it undesirable. The fact that this equilibrium is not exactly $0$ is the effect of migration. A system will be called resilient if the only stable equilibrium that exists is $x_*^+(w)$. In this case the migration rate is enough bring the population back to abundance. In the non-resilient case, when $x_*^0(w)$ is present, an eradicated species cannot return to abundance due to migration.

When the system is away from the region where the bifurcation can happen, our method predicts the equilibrium with very good accuracy. Figure 3a show the typical picture for Erdös-Rényi graphs. Because the second order approximation is indistinguishable from the first in this scale, only the first order approximation is drawn.

*1) Critical Weight:* The first order approximation of the vertex dynamics is given by

$$\dot{x}_i = f(x_i) + w_i g(x_i, e^{\{0\}}(w_{\text{av}})).$$

A saddle-node bifurcation happens also in this dynamics, so it is natural to ask at which is the critical value of $w_i$, i.e. $w_{\text{crit}} \in \mathbb{R}$ such that when $w_i > w_{\text{crit}}$, then there is only one stable equilibrium away from 0 and when $w_i < w_{\text{crit}}$, there are two stable equilibria, one of which is close to 0.

The critical weight, $w_{\text{crit}}$, is a function of $w_{\text{av}}$ since it is a function of $e^{\{0\}}$ and $e^{\{0\}}$ is a function of $w_{\text{av}}$. In Figure 2 we see the graph of $w_{\text{crit}}$ versus $w_{\text{av}}$ for the mutualistic interactions dynamics. Notice that because $e^{\{0\}}$ is discontinuous, $w_{\text{crit}}$ is also discontinuous.

The critical weigh can reveal some basic properties for the dynamics on a nodal level. For example we see in Figure 2 that when when $w_{\text{av}} > w_*^+$, $w_{\text{crit}}$ is almost 0. This implies that if the system on average is in the resilient region, a vertex will also be in the resilient region even if it is very weakly connected to the rest of the network.

Notice that by definition the critical weight is graph-agnostic, since the mean field approximation is graph agnostic. We can use $e^{\{n\}}$, $n > 0$, instead of $e^{\{0\}}$ to calculate the critical weight to get a better estimate. In this case we need to know the distribution of weighted in-degrees of the graph.

*2) Loss of Resilience:* As a demonstration of our method we consider an Erdös-Rényi graph with $30$ vertices and $p = 0.5$ and edge weights uniformly distributed in $[0, 2]$. We consider three cases of perturbation, removal of vertices, removal of edges and uniform reduction of weights. We do Monte Carlo simulations of the perturbations. The results are presented in Figure 3 of the main text.

At each step we apply the perturbation and we compute the 3rd order approximation of the system along with the 3rd order bounds. The point where the lower bound drops close to 0 marks the beginning of our uncertainty interval. The point where the upper bound drops close to 0 marks the end of our uncertainty interval. We also perform a numerical simulation of the system and we compare this with our approximation.

In the case of removal of vertices at each step we remove a random vertex and we keep the largest connected component of the graph if there is more than one, or one at random if we end up with two connected component of equal sizes, until the point where only one vertex remains. We do this operation 20 times and we plot the values of the equilibrium at each vertex. We see that the approximation is very accurate outside the uncertainty interval and the loss of resilience happens in the uncertainty interval.

Similarly in the case of edge removal, at each step we remove a random edge until the graph becomes completely disconnected. We do this 20 times and we plot the values of the equilibrium at each vertex. We see again that the approximation is very accurate outside the uncertainty interval and the loss of resilience

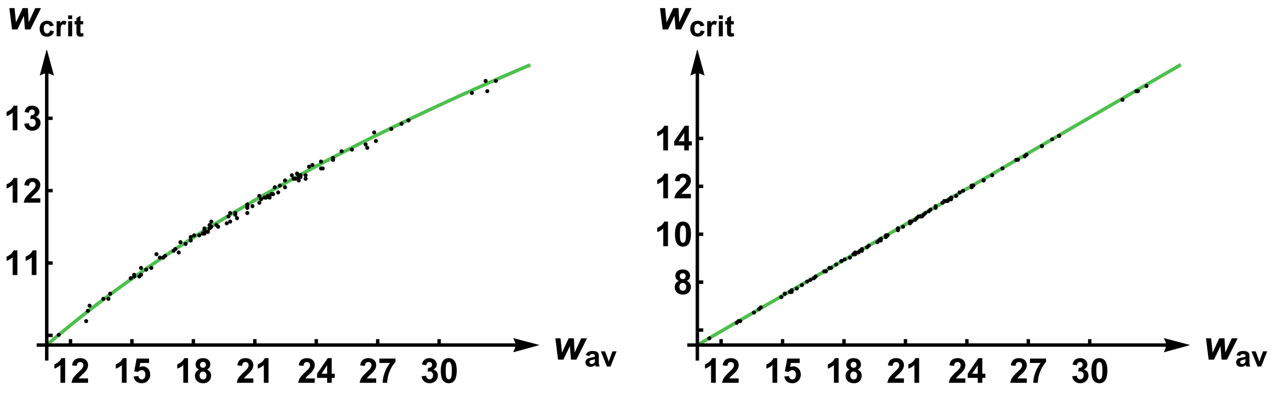


(a) Mutualistic interactions dynamics.

(b) Gene regulatory dynamics.

Fig. 3: The first order approximation of two dynamical systems on the same Erdös-Rényi graph with 100 vertices and $p = 0.2$. The horizontal axis is the weighted in-degree of a vertex, $w_i$, and the vertical axis is the value of the equilibrium at this vertex. The equilibrium computed numerically is shown in black and the blue line is the graph of the function $\chi^{\{1\}}$.

happens in the uncertainty interval.

Finally in the case of weight reduction, we multiply each weight by a factor $r \in [0, 1]$, starting with $r = 1$ and moving to $r = 0$ in $100$ steps. Since this is a deterministic perturbation we do this only once. Once more we plot the values of the equilibrium at each vertex. We see again that the uncertainty interval is useful to determine the region were the loss of resilience will happen.

### *B. Gene Regulatory Networks*

The dynamics in this model is given by

$$f(x) = -Bx^{\alpha},$$
$$g(x, y) = \frac{y^{\beta}}{1 + y^{\beta}},$$

We follow [2] and set $B = 1$, $\alpha = 1$, and $\beta = 2$. By looking at the bifurcation diagram, Figure 4, of equation

$$\dot{x} = -x + w\,\frac{x^{\beta}}{1 + x^{\beta}} \tag{7}$$

we see that a saddle-node bifurcation happens at $w_* = 4$. If $w < w_*$, the only stable equilibrium of the system is $x = 0$. If $w > w_*$ a pair of a stable and an unstable equilibrium appears in the system. The equilibrium $x = 0$ implies cell death and it will be considered undesirable. The positive stable equilibrium corresponds to cell survival. We will call the system resilient if a positive stable equilibrium exists.

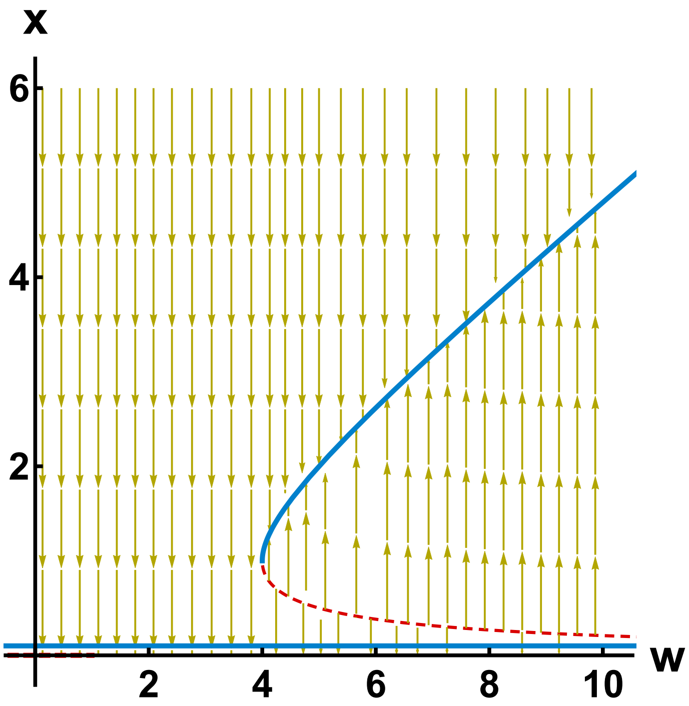


Fig. 4: The bifurcation diagram of (7). The stable equilibria are marked with a solid blue line and the unstable equilibrium is marked with a dashed red line.

The peculiarity of this dynamics is that the vertex dynamics is trivial,

$$\dot{x}_i = f(x) + w_i g(x_i, e) = -x_i + w_i \frac{e^\beta}{1+e^\beta}. \tag{8}$$

The second term of the right-hand side is constant so the equilibrium is just $x = w_i e^\beta/(1+e^\beta)$. Because of this the idea of critical weight that we discussed in the previous section cannot be applied here, as no bifurcation happens in (8). For the same reason the first order approximation is basically the best we can do in this dynamics. However, as Figure 3b shows, the first order approximation is already very accurate. A final implication of the triviality of (8) is that once the mean field approximation becomes $0$, then the next approximations and bounds all become $0$.

*1) Loss of Resilience:* As in the case of mutualistic interactions we demonstrate the usefulness of our method on a Erdös-Rényi graph with $30$ vertices and $p = 0.5$ and edge weights uniformly distributed in $[0, 2]$. We consider the same types of perturbation, i.e. removal of vertices, removal of edges and uniform reduction of weights. We do Monte Carlo simulations of the perturbations. The results are presented in Figure 4 of the main text.

At each step we compute the $1$st order approximation of the system along with the bounds, as explained above in this dynamics this is the can do. Because of the peculiarity of this system, the lower bound drops

gradually to 0 and it never becomes 0 before the whole network collapses. The actual critical value below which activity is assumed to have ceased depends on the biological system that is being considered. Here we have chosen $0.1$ as this critical value and we mark the region where the lower bound has dropped below it. The upper bound performs a more dramatic drop so we can use it as before. We also perform a numerical simulation of the system and compare this with our approximation. The results are shown in Figure 4 of the main text.

We consider three cases of perturbation, removal of vertices, removal of edges and uniform reduction of weights. These are performed the same way they were performed in the case of ecological networks. We see that in this system the loss of resilience happens with a less dramatic drop compared to the mutualistic interaction, yet it happens in the uncertainty interval we compute.

In the case of uniform weight reduction, because of the peculiarity of gene regulatory dynamics, once the mean field approximation becomes $0$ everything collapses to $0$. This is the reason that the uncertainty interval becomes very narrow and just misses the actual loss of resilience. We could get a wider uncertainty interval by repeating the process using the uniform mean for the mean field approximation. This would make the interval large enough that it would include the point where the loss of resilience happens. However, in order to keep the exposition clean and consistent we decided against this idea.

### *C. Counter-Intuitive Dynamics*

The dynamics studied in the previous sections follow the conventional wisdom, where a more connected network is always more resilient. This happens because the function $G(x,y)$ was in both cases strictly increasing with respect to $y$. We will see that we can define dynamics where even though we can make a vertex resilient by adding more edges to it, we can also have the same result by removing edges from the network. This phenomenon is presented in Figure 6 of the main text. We will use the dynamics defined by

$$\begin{aligned} f(x) &= \frac{1}{10} + x\,(1-x)\left(\frac{x}{5}-1\right), \\ g(x,y) &= 15\frac{x^2 y}{y^2+1}. \end{aligned} \tag{9}$$

For this dynamics we used the same $f$ as in mutualistic interaction in ecological networks but we changed the function $g$. Figure 9 shows the bifurcation diagram of the dynamics (9). Notice the similarities with the bifurcation diagram of the dynamics of ecological networks in Figure 1. We will keep the convention

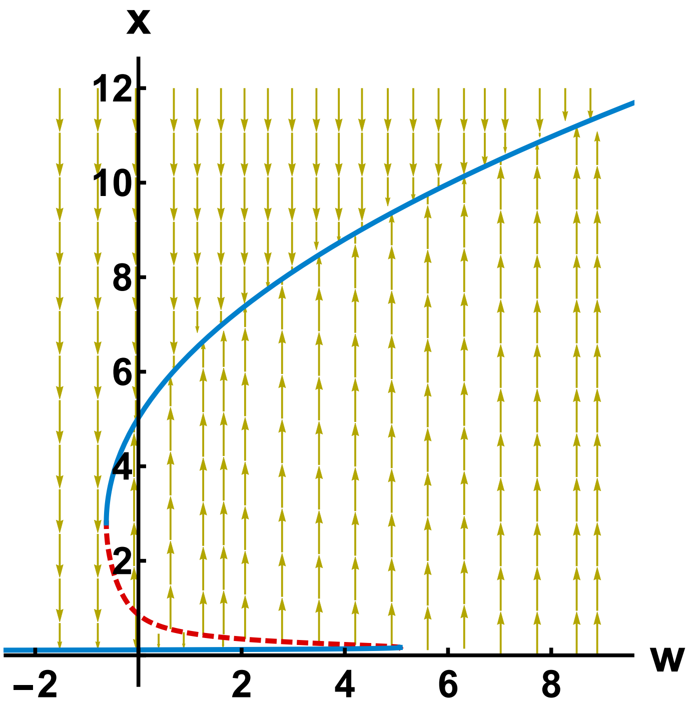


Fig. 5: The bifurcation diagram of (9). The stable equilibria are marked with a solid blue line and the unstable equilibrium is marked with a dashed red line.

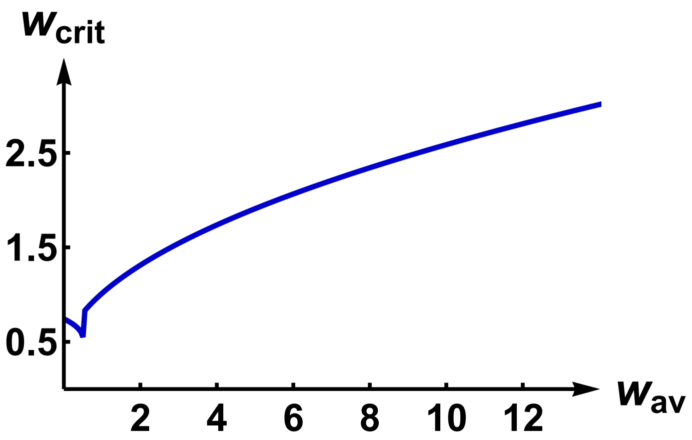


Fig. 6: The graph of graph of the critical degree function for the dynamics 9.

of section II-A and we will say that a vertex is resilient if there is only one stable equilibrium which is away from 0.

As an example we use an undirected graph with 30 vertices, 121 edges and edge weights equal to 1. For this graph, the mean weighted degree is 8.9 and the average degree is 8. The weighted degree of vertex 11 is 2, which is the minimum. In Figure 6 we see the graph of the critical degree function for the dynamics chosen here. When $w_{\text{av}} = 8$ the critical degree is approximately $2.31$ and $w_{\text{av}} = 8.8$ the critical degree is approximately $2.32$. Because of this we expect that vertex 11 is not resilient. Numerical

simulation proves that our prediction was correct.

If we add a new edge at vertex 11, we will bring the its degree to 3 which is in the resilient region. Again we can check and see that numerical simulation proves us correct.

However, by looking at Figure 6 we can see that there is an alternative way to make vertex 11 resilient. We can prune edges from our network that connect to high degree vertices and bring the average degree down. We remove 46 edges and bring the average degree down to $5$ and the weighted average degree to $5.26$, these correspond to critical degrees of $1.9$ and $1.95$ and it predicts that vertex 11 is not resilient. Numerical simulation proves this prediction correct.

### *D. Approximation of equilibria of different graph models*

In the main article we have shown how our method works on a Bernoulli graph with 30 vertices and average degree approximately 15. Here we provide 5 more examples all graphs with 30 vertices, a Bernoulli graph with average degree approximately 7.5, two Watts-Strogatz graphs with average degrees 8 and 14 and two Barabasi-Albert graphs with average degrees 15 and approximately 7.

The average edge weight was 1 when the average degree was approximately 15 and 2 when the average degree was approximately 7.5. This was done to ensure that the dynamics on the initial graph is resilient. The Bernoulli graphs were chosen to be connected. The Watts-Strogatz graph were generated with parameter $\beta = 0.1$. For high $\beta$ the generated graph distribution becomes similar to the Bernoulli graph distribution.

## References

[1] M. E. J. Newman, *Networks: an introduction*. Oxford; New York: Oxford University Press, 2010.

[2] J. Gao, B. Barzel, and A. Barabasi, “Universal resilience patterns in complex networks,” *Nature*, vol. 530, 2016.

[3] S. Richard, “Varga: Geršgorin and his circles,” 2004.

[4] J. Szücs, M. Levi, and V. Arnold, *Geometrical Methods in the Theory of Ordinary Differential Equations*, ser. Grundlehren der mathematischen Wissenschaften. Springer New York, 2012. [Online]. Available: https://books.google.co.uk/books?id=nejlBwAAQBAJ

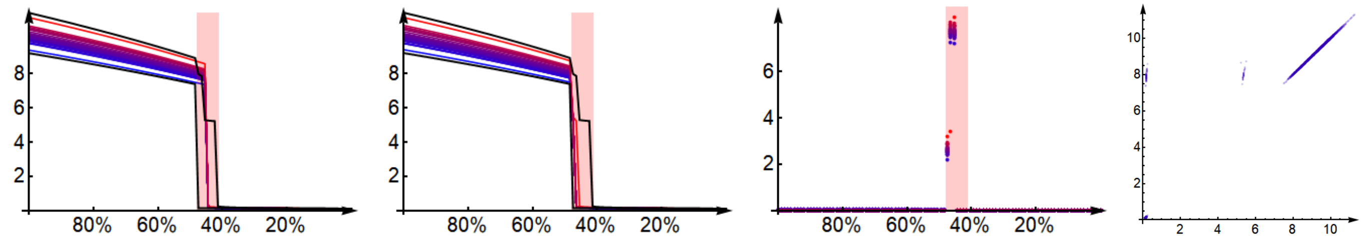


(a) (b) (c) (d)

Fig. 7: Resilience loss from coupling weight loss for dynamics on a Watts–Strogatz graph with mean degree 14. (a) Numerical simulation of the equilibrium. (b) Approximation of the equilibrium. (c) The difference between numerical simulation and approximation. (d) Approximation plotted against numerical simulation.

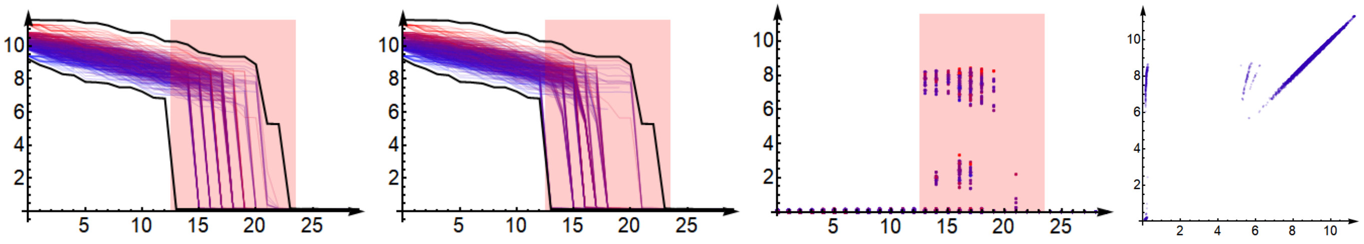

(a) (b) (c) (d)

Fig. 8: Resilience loss from random node removal for dynamics on a Watts–Strogatz graph with mean degree 14. (a) Numerical simulation of the equilibrium. (b) Approximation of the equilibrium. (c) The difference between numerical simulation and approximation. (d) Approximation plotted against numerical simulation.

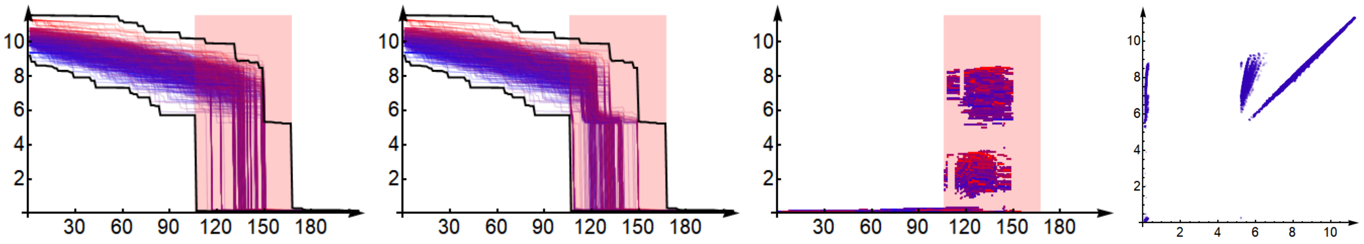

(a) (b) (c) (d)

Fig. 9: Resilience loss from random edge removal for dynamics on a Watts–Strogatz graph with mean degree 14. (a) Numerical simulation of the equilibrium. (b) Approximation of the equilibrium. (c) The difference between numerical simulation and approximation. (d) Approximation plotted against numerical simulation.

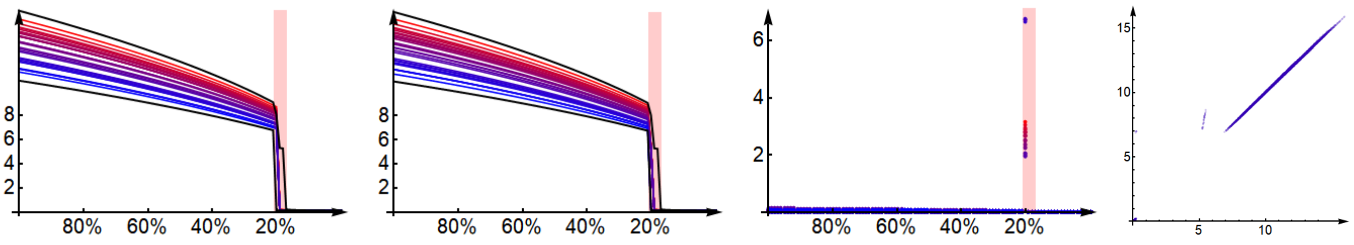

(a) (b) (c) (d)

Fig. 10: Resilience loss from coupling weight loss for dynamics on a Barabási–Albert graph with mean degree 15. (a) Numerical simulation of the equilibrium. (b) Approximation of the equilibrium. (c) The difference between numerical simulation and approximation. (d) Approximation plotted against numerical simulation.

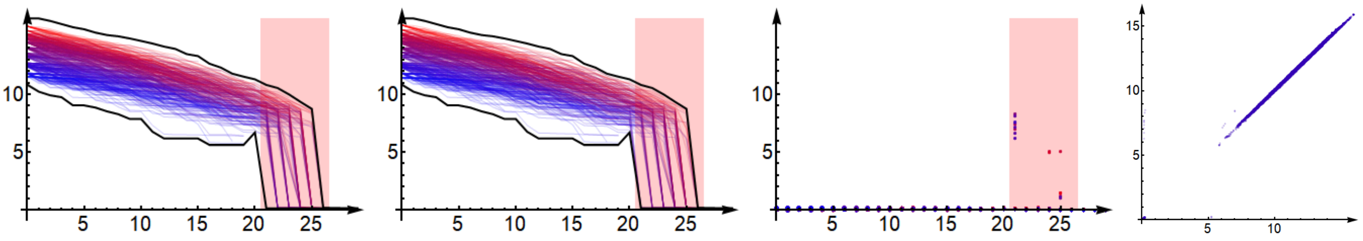

(a) (b) (c) (d)

Fig. 11: Resilience loss from random node removal for dynamics on a Barabási–Albert graph with mean degree 15. (a) Numerical simulation of the equilibrium. (b) Approximation of the equilibrium. (c) The difference between numerical simulation and approximation. (d) Approximation plotted against numerical simulation.

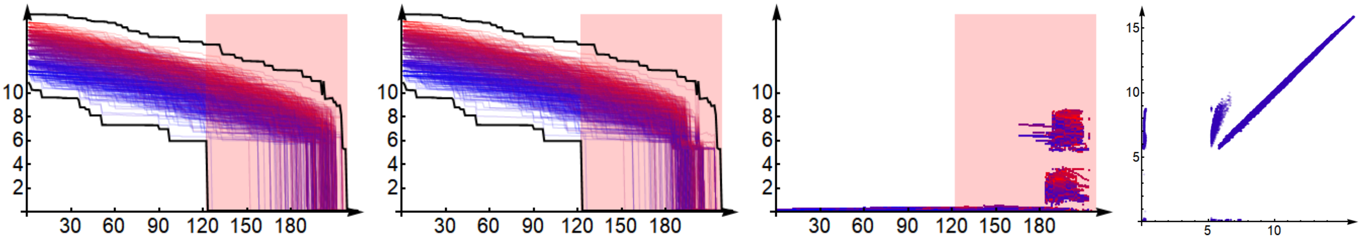

(a) (b) (c) (d)

Fig. 12: Resilience loss from random edge removal for dynamics on a Barabási–Albert graph with mean degree 15. (a) Numerical simulation of the equilibrium. (b) Approximation of the equilibrium. (c) The difference between numerical simulation and approximation. (d) Approximation plotted against numerical simulation.

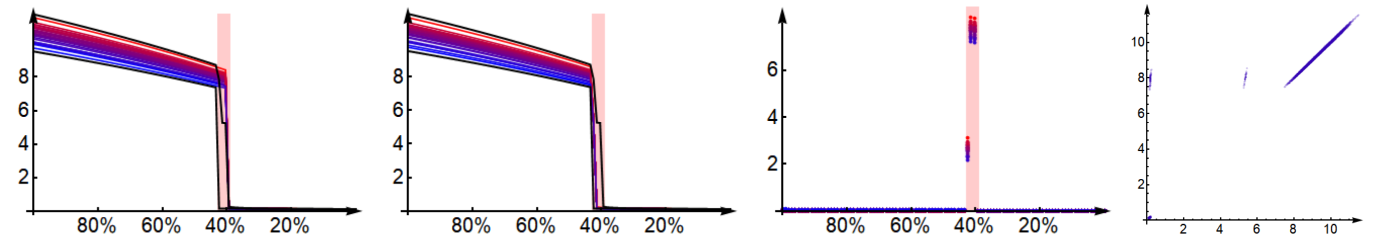


(a) (b) (c) (d)

Fig. 13: Resilience loss from coupling weight loss for dynamics on a Watts–Strogatz graph with mean degree 8. (a) Numerical simulation of the equilibrium. (b) Approximation of the equilibrium. (c) The difference between numerical simulation and approximation. (d) Approximation plotted against numerical simulation.

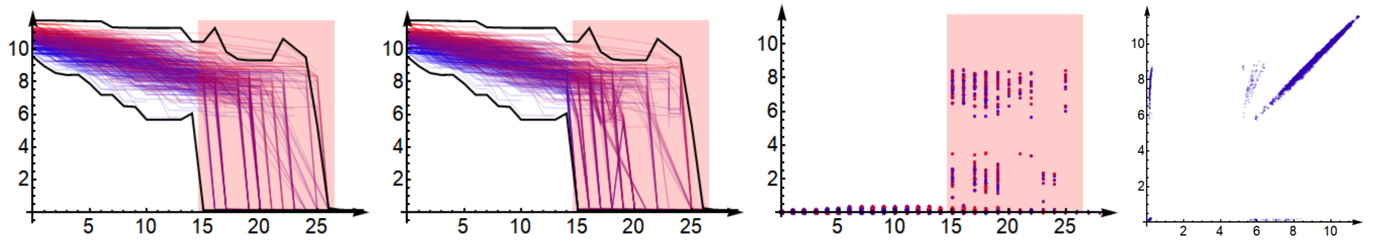


(a) (b) (c) (d)

Fig. 14: Resilience loss from random node removal for dynamics on a Watts–Strogatz graph with mean degree 8. (a) Numerical simulation of the equilibrium. (b) Approximation of the equilibrium. (c) The difference between numerical simulation and approximation. (d) Approximation plotted against numerical simulation.

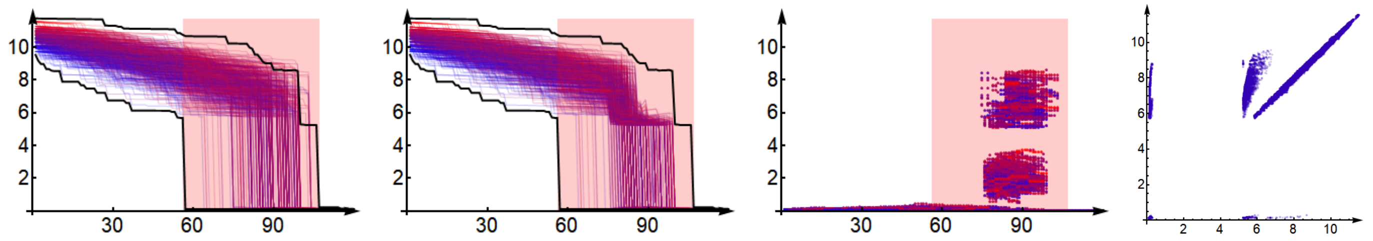


(a) (b) (c) (d)

Fig. 15: Resilience loss from random edge removal for dynamics on a Watts–Strogatz graph with mean degree 8. (a) Numerical simulation of the equilibrium. (b) Approximation of the equilibrium. (c) The difference between numerical simulation and approximation. (d) Approximation plotted against numerical simulation.

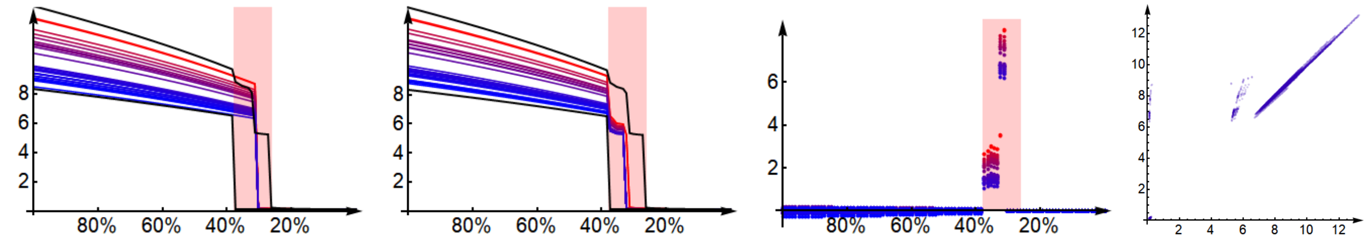


(a) (b) (c) (d)

Fig. 16: Resilience loss from coupling weight loss for dynamics on a Barabási–Albert graph approximately 7.5. (a) Numerical simulation of the equilibrium. (b) Approximation of the equilibrium. (c) The difference between numerical simulation and approximation. (d) Approximation plotted against numerical simulation.

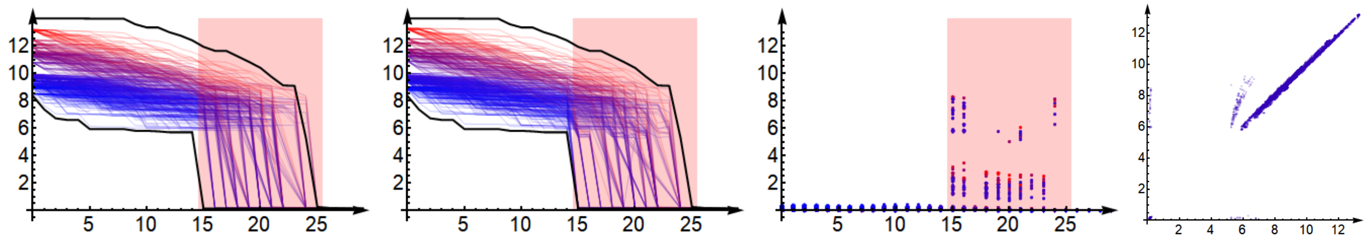


(a) (b) (c) (d)

Fig. 17: Resilience loss from random node removal for dynamics on a Barabási–Albert graph approximately 7.5. (a) Numerical simulation of the equilibrium. (b) Approximation of the equilibrium. (c) The difference between numerical simulation and approximation. (d) Approximation plotted against numerical simulation.

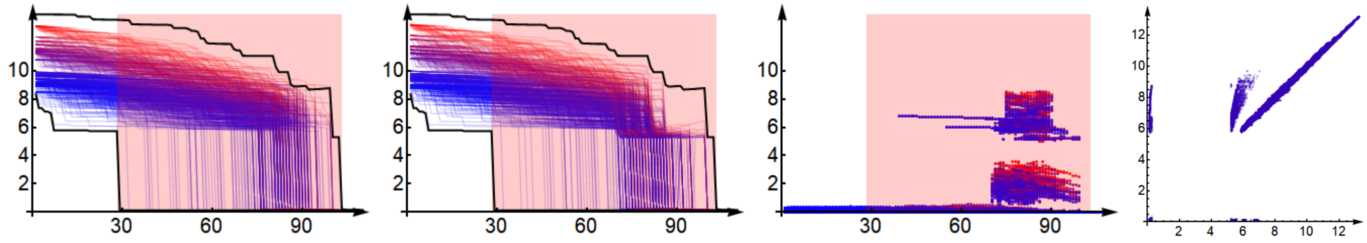


(a) (b) (c) (d)

Fig. 18: Resilience loss from random edge removal for dynamics on a Barabási–Albert graph approximately 7.5. Notice that in this case some nodes collapse early. This happens because there are nodes with small degree and they happen to have all their edges removed. (a) Numerical simulation of the equilibrium. (b) Approximation of the equilibrium. (c) The difference between numerical simulation and approximation. (d) Approximation plotted against numerical simulation.

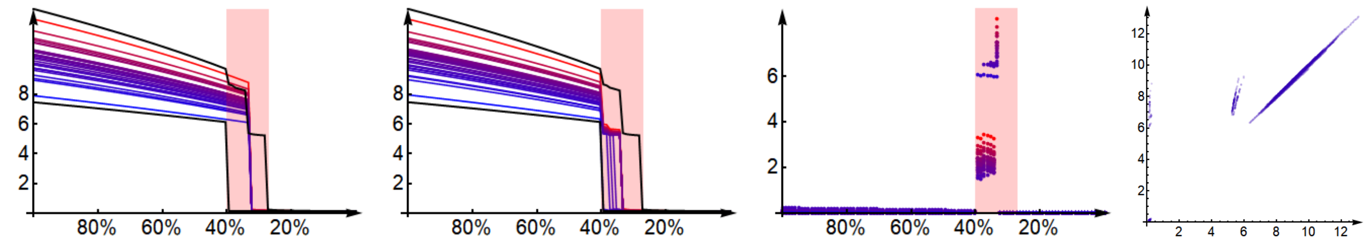


(a) (b) (c) (d)

Fig. 19: Resilience loss from coupling weight loss for dynamics on a Bernoulli graph approximately 7. (a) Numerical simulation of the equilibrium. (b) Approximation of the equilibrium. (c) The difference between numerical simulation and approximation. (d) Approximation plotted against numerical simulation.

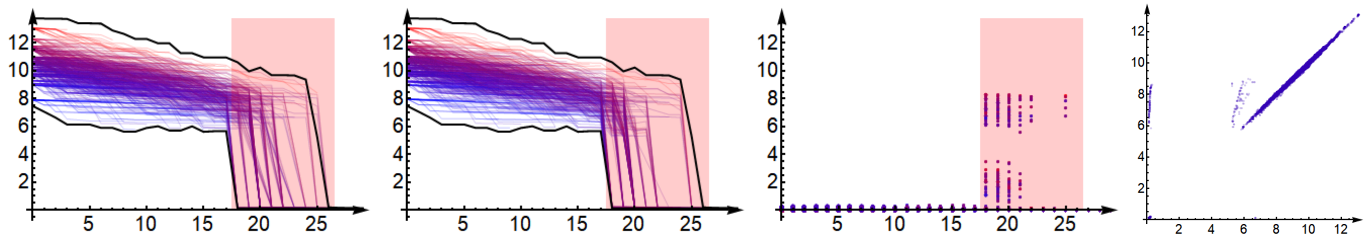


(a) (b) (c) (d)

Fig. 20: Resilience loss from random node removal for dynamics on a Bernoulli graph approximately 7. (a) Numerical simulation of the equilibrium. (b) Approximation of the equilibrium. (c) The difference between numerical simulation and approximation. (d) Approximation plotted against numerical simulation.

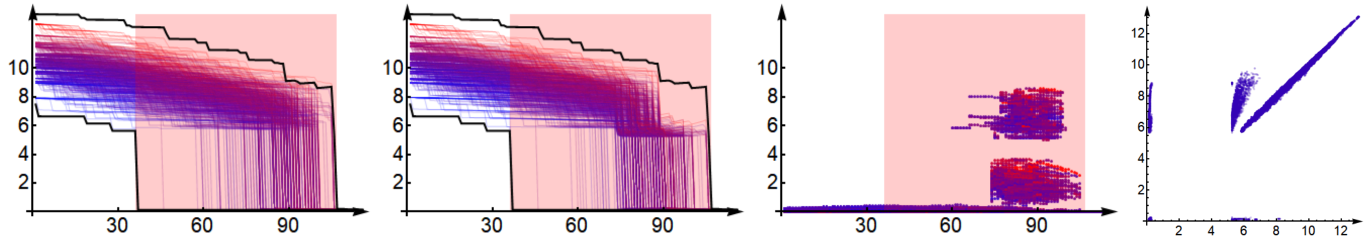


(a) (b) (c) (d)

Fig. 21: Resilience loss from random edge removal for dynamics on a Bernoulli graph approximately 7. Notice that similarly to the Barabási—Albert graph, low degree nodes get detached from the network and they collapse. (a) Numerical simulation of the equilibrium. (b) Approximation of the equilibrium. (c) The difference between numerical simulation and approximation. (d) Approximation plotted against numerical simulation.
